# A Robust Drug–Target Interaction Prediction Framework with Capsule Network and Transfer Learning

**DOI:** 10.3390/ijms241814061

**Published:** 2023-09-14

**Authors:** Yixian Huang, Hsi-Yuan Huang, Yigang Chen, Yang-Chi-Dung Lin, Lantian Yao, Tianxiu Lin, Junlin Leng, Yuan Chang, Yuntian Zhang, Zihao Zhu, Kun Ma, Yeong-Nan Cheng, Tzong-Yi Lee, Hsien-Da Huang

**Affiliations:** 1 School of Medicine, The Chinese University of Hong Kong, Shenzhen, Longgang District, Shenzhen 518172, China; yixianhuang@link.cuhk.edu.cn (Y.H.); yigangchen@link.cuhk.edu.cn (Y.C.); junlinleng@link.cuhk.edu.cn (J.L.);; 2 Warshel Institute for Computational Biology, The Chinese University of Hong Kong, Shenzhen, Longgang District, Shenzhen 518172, China; lantianyao@link.cuhk.edu.cn (L.Y.); yuanchang@link.cuhk.edu.cn (Y.C.);; 3 Institute of Bioinformatics and Systems Biology, Department of Biological Science and Technology, National Yang Ming Chiao Tung University, Hsinchu 300, Taiwan; tom.cheng0911@gmail.com (Y.-N.C.);

**Keywords:** drug–target interactions, bidirectional encoder representations from transformers, transfer learning, message-passing neural networks, capsule network

## Abstract

Drug–target interactions (DTIs) are considered a crucial component of drug design and drug discovery. To date, many computational methods were developed for drug–target interactions, but they are insufficiently informative for accurately predicting DTIs due to the lack of experimentally verified negative datasets, inaccurate molecular feature representation, and ineffective DTI classifiers. Therefore, we address the limitations of randomly selecting negative DTI data from unknown drug–target pairs by establishing two experimentally validated datasets and propose a capsule network-based framework called CapBM-DTI to capture hierarchical relationships of drugs and targets, which adopts pre-trained bidirectional encoder representations from transformers (BERT) for contextual sequence feature extraction from target proteins through transfer learning and the message-passing neural network (MPNN) for the 2-D graph feature extraction of compounds to accurately and robustly identify drug–target interactions. We compared the performance of CapBM-DTI with state-of-the-art methods using four experimentally validated DTI datasets of different sizes, including human (*Homo sapiens*) and worm (*Caenorhabditis elegans*) species datasets, as well as three subsets (new compounds, new proteins, and new pairs). Our results demonstrate that the proposed model achieved robust performance and powerful generalization ability in all experiments. The case study on treating COVID-19 demonstrates the applicability of the model in virtual screening.

## 1. Introduction

Drug–target interaction, or DTI, refers to the recognition of the interaction between drugs and the protein targets that may result in illness in humans. Such binding typically causes the target protein to alter its physicochemical characteristics and malfunction as a result. Discovering and understanding these interactions can not only assist explain the mechanism of many currently available medications, but also be employed for drug repositioning and side effect forecasting [1]. Therefore, DTI prediction is one of the most significant research areas during the drug development process [2]. Wet lab experiments are the most reliable and effective method for determining whether a drug interacts with its target, but they are time-consuming and resource-intensive [3]. To alleviate this problem, several computational methods were proposed to speed up the development of new drugs and reduce costs.

Structure-based methods and ligand-based methods are the two main existing computational approaches for identifying drug–target interactions [4]. In structure-based strategies such as molecular docking, the three-dimensional (3D) structures of proteins and chemical compounds are utilized to explore potential binding poses at the atom level and identify binding affinities [5]. Nevertheless, the great computational difficulty of solving such 3D structures and the scarcity of small molecules and proteins with known 3D structures limits the scope of these approaches, even though they yielded adequate biological interpretation and somewhat attractive predictive performance [6].

Ligand-based methods, including machine learning-based methods and deep learning-based methods, require less computational resources than structure-based methods because they rely on only one or two-dimensional sequence information of proteins and chemical compounds [1,7]. As for the machine learning-based method, it extracts discriminative biological features for chemical compounds and target proteins in a drug–target pair and feeds the extracted features into a machine learning model such as random forest, logistic regression, support vector machine, and other kernel-based methods to determine whether the drug and the target protein will interact or not. Wang et al. use the structure similarity profile of drugs and the sequence similarity profile of proteins to encode a given drug–protein pair to obtain the feature vector that is inputted into support vector machine (SVM) to predict drug–protein interactions [8]. Tabei et al. employ an improved min-wise hashing algorithm to construct new compact fingerprints for compound–protein pairs and adopts linear SVM to make a large-scale prediction of compound–protein interactions [9]. Yu et al. extract conserved patterns from proteins and their corresponding ligands and then construct a systematic approach based on Random Forest (RF) and SVM to predict drug–target interaction [10].

Although machine learning-based methods are highly generalizable and sequence-sensitive (the arrangement and order of elements within the data sequences such as drug SMILE sequences and target protein sequences will affect the predictions or decisions of the model), they are constrained by excessive reliance on hand-crafted, expert knowledge-based features [1,11]. The aforementioned restrictions might be overcome via deep learning techniques, namely end-to-end differential models. In fact, despite having a lot of parameters, they may automatically learn the characteristics and invariances of provided data and offer a good generalization. For example, Öztürk et al. propose a convolutional neural-based method called DeepDTA to predict the binding affinities of protein–ligand interactions which employ CNN blocks to learn representations from the raw protein sequences and SMILES strings and combine these representations to feed into a fully connected layer block [12]. MolTrans, built by Huang et al., adopts frequent consecutive sub-sequence (FCS) mining to extract fit-sized sub-structures for both protein and drug, which is further processed through an augmented transformer-embedding module to predict drug–target interaction [13]. Cheng et al. developed a bidirectional encoder–decoder structure named IIFDTI to extract interactive features of substructures between drugs and targets and to input into fully connected dense layers in downstream tasks for predicting DTIs [14]. Chatterjee et al. propose the AI-Bind to overcome the limitations of current deep learning models in predicting novel drug–target interactions. AI-Bind includes two layers corresponding to binding and non-binding annotations between proteins and ligands. Positive and negative link probabilities are determined by entropy maximization and are used to estimate the conditional probability in transductive, semi-inductive, and inductive conditions [15]. Despite the progress made in DTI prediction models, they still have limitations. For instance, some methods lack experimentally verified negative datasets [16], while others are restricted to specific datasets, making them less robust due to inaccurate molecular feature representation and ineffective DTI classifiers [9]. Such limitations can result in overfitting the training data, causing the models to be adapted to only specific cohorts of data and perform poorly when applied to unseen drug–target pairs. Inaccurate molecular feature representation can lead to issues such as missing or inaccurate information, introducing noise and inconsistency into the model. Ineffective DTI classifiers may produce inaccurate predictions by failing to capture the categorical properties of input features, leading to false-positive and false-negative predictions.

Taking into account the limitations of the existing models, a novel robust capsule-based prediction model was constructed to accurately identify DTIs on four experimentally validated DTI datasets of different sizes, including human (*Homo sapiens*) and worm (*Caenorhabditis elegans*) species datasets, as well as three subsets (new compounds, new proteins, and new pairs). Briefly, we use the simplified molecular input line system (SMILES) string of the compounds, a line notation that uses predefined rules to describe the structure of a compound sequentially, and the target sequence of the compounds as input. Our model is made up of three major modules. The first module is a 2-dimensional (2-D) graph feature encoder, which converts SMILES into an atomic graph and employs the message-passing neural network (MPNN) to extract the graph’s high-order structures as well as semantic relations. Because pharmaceuticals are primarily graph-structured, with atoms as nodes and bonds as edges, the atomic graph representation is natural and sensible. The second module uses ProtBert [17] to vectorize protein sequences, rather than using the physicochemical properties of amino acids to encode proteins and perform clustering. This approach maps each word (amino acids are considered as words) to a potential vector space where geometric relationships can be applied to characterize semantic relationships. The last module is to add structures called “capsules” as a better model of hierarchical relationships. In this step, we apply a new activation function called ‘squash’, and another process widely known as dynamic routing between capsules. The robust representations generated by this module are highly discriminative in distinguishing between DTIs and non-DTIs. In comparison to former studies, our novelty is outlined as follows:

- To overcome the limitation of other studies in which negative DTI data are randomly selected from unknown drug–target pairs, we established two experimentally validated datasets.
- Protein sequences are treated as natural language and are vectorized by the state-of-the-art ProtBert model through transfer learning, while drug molecules are transformed by MPNN. Both encoding approaches represent protein targets and drug molecules more precisely.
- The proposed capsule network-based DTI prediction model describes the internal hierarchical representation of features. It outperforms other existing established SOTA DTI prediction tools on seven experimentally validated DTI datasets with varying amounts of data, including human (*Homo sapiens*) and worm (*Caenorhabditis elegans*) species datasets.

## 2. Results

### 2.1. Effectiveness of BERT Module, MPNN Module, and Capsule Network Module

**Figure 1:**
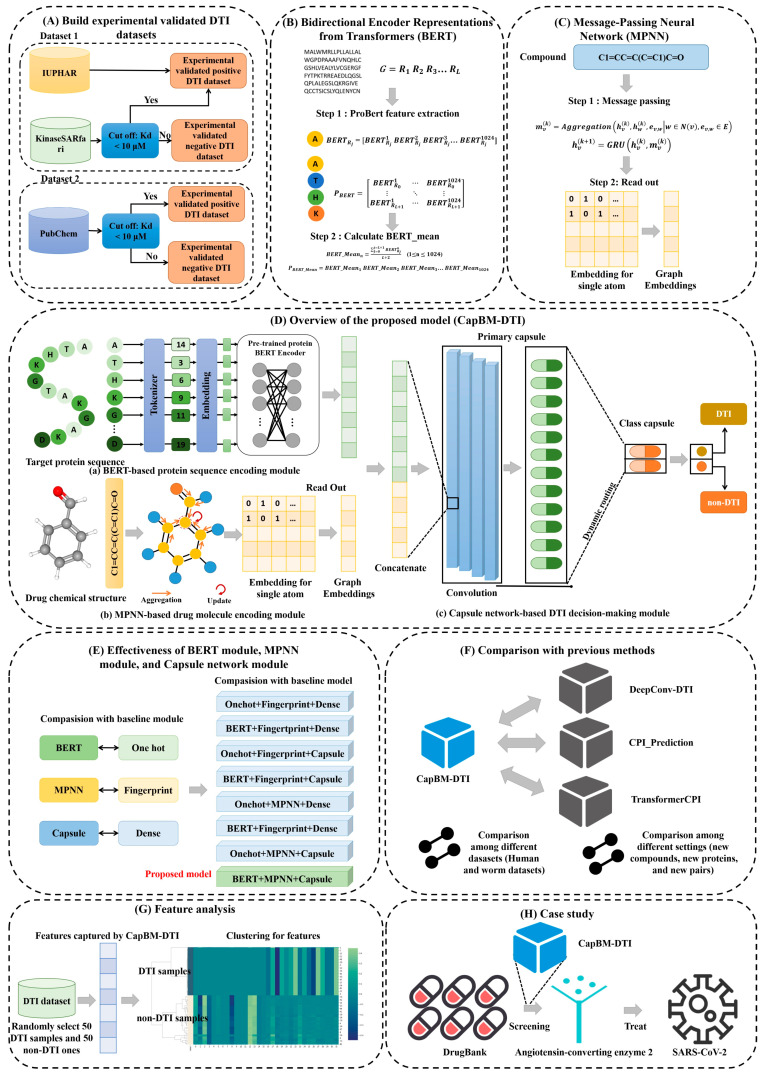
The schematic of this study. (**A**) Build two experimentally validated DTI datasets. (**B**) The principles of feature extraction from protein sequence using bidirectional encoder representations from transformers (BERT). (**C**) The principles of feature extraction from drug molecules using the message-passing neural network (MPNN). (**D**) Overview of the proposed model (CapBM-DTI). It has three modules: (**a**) BERT-based protein sequence encoding module, (**b**) MPNN-based drug molecule encoding module, and (**c**) the capsule network-based DTI decision-making module. (**E**) Effectiveness of BERT module, MPNN module, and capsule network module. Seven baseline models with different protein sequence features, drug molecule features, and DTI decision-making modules were selected as baselines to investigate the effectiveness of the BERT module, MPNN module, and capsule network module in the proposed model. (**F**) Comparison with previous SOTA methods among different datasets (human and worm datasets) and different settings (new compounds, new proteins, and new pairs). (**G**) Feature analysis of DTI-related features extracted by the proposed CapBM-DTI. (**H**) Case study of drug repurposing to treat COVID-19.

In this study, seven models with different protein sequence features, drug features, and DTI decision-making modules were selected as baselines to investigate the effectiveness of the BERT module, MPNN module, and capsule network module in the proposed model (Figure 1E). Specifically, one-hot encoding and BERT were used for protein sequence feature extraction, while fingerprint and MPNN were employed for feature extraction of drug structure (SMILES). As for the DTI decision-making module, the dense layer and the capsule layer were adopted to differentiate the DTI class and the non-DTI class. Overall, there are seven baseline models for comparison; that is, BERT + MPNN + dense model, BERT + fingerprint + capsule model, BERT + fingerprint + dense model, one-hot + MPNN + capsule model, one-hot + MPNN + dense model, one-hot + fingerprint + capsule model, and one-hot + fingerprint + dense model.

**Figure 2:**
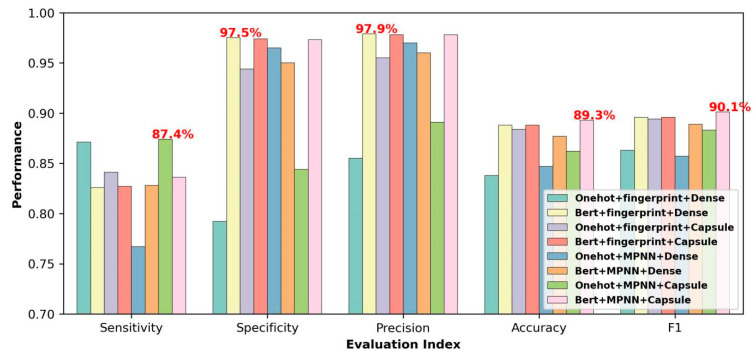
Comparison of performances between our models and baseline models on Dataset 1.

The performance of the proposed CapBM-DTI model was compared with the above seven baseline models on Dataset 1 (the reason for choosing Dataset 1 is that Dataset 1 is a medium-sized expert-curated dataset, and its comparison results are representative), and the results are shown in Figure 2 and Table S3. For the model training and independent testing, it was randomly assigned according to a ratio of 8:2. This means that the data consisted of 13,302 positive and 9414 negative samples, randomly selected for model training, and the rest of the data (3325 positives and 2354 negative samples) were used for external (independent) testing. The training and independent samples did not overlap. The model built in this paper outperforms all baseline models in terms of accuracy and F1 score. Specifically, this model achieved an accuracy of 89.3% and an F1 score of 90.1%. Although the other metrics (sensitivity, specificity, and precision) are not the best compared to the baseline models, the highest values of accuracy and F1 score are enough to prove that the proposed model is the most effective and powerful because accuracy is the most widely used metric to evaluate a model performance considering all correctly predicted cases whether positive or negative, and F1 score is a very robust evaluation metric that works great for many classification problems due to taking both false positives and false negatives into account.

**Figure 3:**
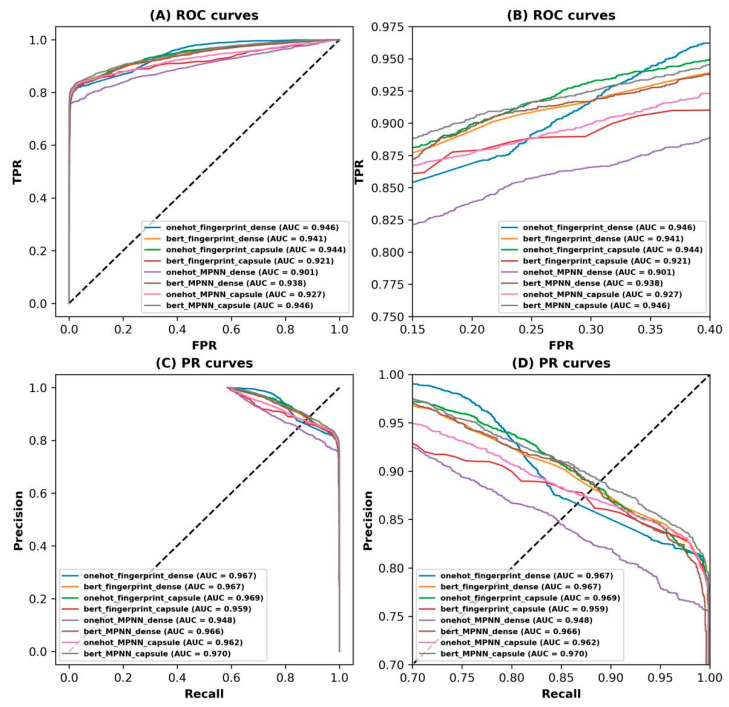
ROC and PR curves for our models and baseline models on Dataset 1. (**A**) ROC curves. (**B**) ROC curves for partially enlarged. (**C**) PR curves. (**D**) PR curves for partially enlarged.

To further intuitively compare the proposed CapBM-DTI model with other baseline models, ROC and PR curves were utilized to compare the model with other baselines as illustrated in Figure 3 and Table S3. It can be observed that this model achieved the highest auROC value of 0.946 and auPRC value of 0.97 for the independent test set compared with the baseline models, showing that the model exhibited more excellent predictive ability than baseline models in identifying DTI. To compare the performance of the models graphically, t-distributed stochastic neighbor embedding (t-SNE) visualizations of the proposed model and seven baseline models are shown in Figure S2.

**Figure 4:**
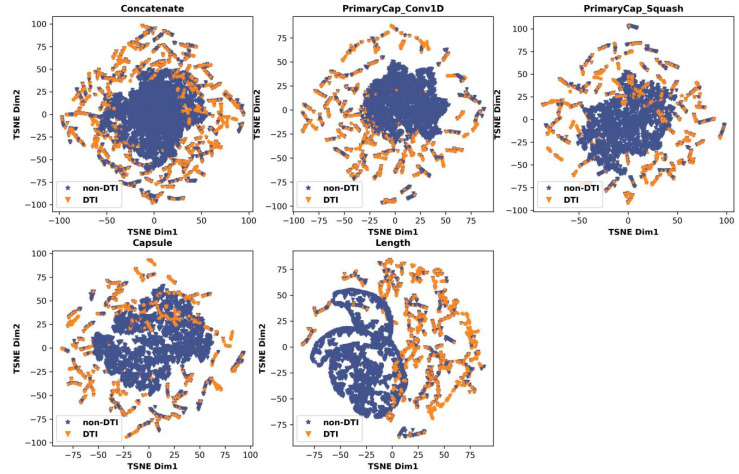
t-SNE visualizations of different layers, using Dataset 1. With these images, it can be seen that the DTI (yellow triangles) and non-DTI (blue stars) are easily distinguishable through the length layer compared to the concatenate layer concatenating target protein and drug molecule features from the BERT layer and MPNN layer.

In addition to the involvement of the BERT layer and the MPNN layer effectively extracting the latent feature of target proteins and drug molecules, respectively, the important reason why the CapBM-DTI model performs well in identifying DTI in the given dataset is the participation of the capsule layer by extracting internal hierarchical representation of concatenated features of protein targets and drug molecules. To visualize the feature extraction process of each layer of the capsule network (PrimaryCap_Conv1D layer, PrimaryCap_Squash layer, capsule layer, and length layer), the features captured by the layers were displayed in scatter 2D space by computed t-SNE. In Figure 4, the yellow triangles and blue stars denote the DTIs and non-DTIs, respectively. With these images, it can be seen that the DTI and non-DTI are more easily distinguishable along with each layer of the capsule layer processes the features from concatenate layer concatenating target protein and drug molecule features from the BERT layer and MPNN layer.

### 2.2. Performance and Generalization Comparison with Existing SOTA Predictors

To further demonstrate the power of the CapBM-DTI predictor, we compared it with several existing methods (Figure 1F). We selected three state-of-the-art (SOTA) deep learning models (i.e., DeepConv-DTI [18], CPI_prediction [19], TransformerCPI [20], and IIFDTI [14]) for comparisons on four datasets (the number of training data: the number of test data = 8:2 in each dataset) in Table 1. The reason why we choose these three methods is that they use experimentally validated datasets, so it is more fair to compare our model with them. Specifically, DeepConv-DTI uses the experimentally validated independent test sets collected from Pubchem and KinaseSafari to test the model while CPI_prediction, TransformerCPI, and IIFDTI use Dataset 3 and Dataset 4 to train and test the models. The comparative results (the scores of accuracy, F1 score, AUC, and AUPR obtained by these three predictors) are illustrated in Table 2 and the best results are shown in bold. As shown in Table 2, the results show that CapBM-DTI obtains the best performance amongst all competing methods in 4 Datasets except the AUC score in Dataset 2, the AUPR score in Dataset 3, and the accuracy score in Dataset 4. Importantly, CapBM-DTI also exhibited excellent performance in the cross-species Dataset 4 (*C. elegans*), proving the strong generalization ability of the model.

In order to further prove the strong predictive performance and generalization ability of the model, we compared the proposed method with the previous model in terms of three settings (new compounds, new proteins, and new pairs; “new” refers to the test set containing unseen items compared to the train set) from Dataset 3 (the reason we choose Dataset 3 for subset splitting is that Dataset 3 is a benchmark dataset for humans, so the split subsets are more comparable). The detailed statistics and definitions of the new compound subset, new protein subset, and new pairs subset are shown in Table 3. As shown in Table 4, the proposed model exhibits superior performance in all three tasks compared to previous predictors except the AUC score in the new compounds dataset and new pair dataset.

In summary, the proposed CapBM-DTI achieves a competitive or better performance than three SOTA predictors in all settings, proving its superior generalization ability and robust performance.

### 2.3. Feature Analysis

**Figure 5:**
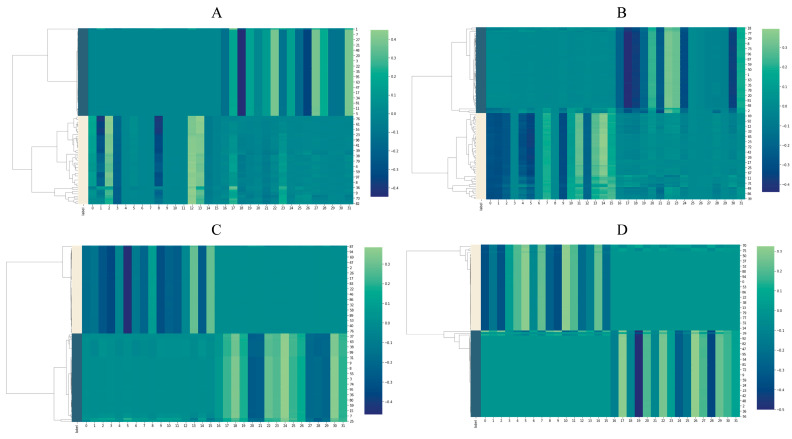
The clustering analysis chart of features obtained by CapBM-DTI on 4 Datasets. (**A**) Dataset 1. (**B**) Dataset 2. (**C**) Dataset 3. (**D**) Dataset 4.

Discriminative features are the key to building a powerful computational classifier. When compared with other existing methods, the proposed CapBM-DTI has two main advantages: (i) integrating BERT and MPNN to extract the feature of protein targets and drug molecules more precisely, and (ii) using the capsule network to extract an internal hierarchical representation of concatenated features of protein targets and drug molecules. The latent and DTI-related features, i.e., DTI and non-DTI, have distinctly different characteristics, and are constructed with 32 features in our capsule networks layer. To highlight the discriminative power of DTI-related features extracted by the proposed CapBM-DTI, we randomly selected 50 DTI samples and 50 non-DTI ones on four datasets (Dataset 1, Dataset 2, Dataset 3, and Dataset 4) and perform a clustering analysis of the features on each dataset (Figure 1G). The clustering results are presented in Figure 5. As shown in Figure 5, we can easily see on each dataset that: (i) DTI and non-DTI samples are clustered into two distinct sub-trees, and (ii) the samples of the DTI class or non-DTI class tend to show similar values of DTI-related features. These results demonstrate that the features extracted by the proposed CapBM-DTI can accurately capture the DTI-related characteristics.

### 2.4. Case Study of Drug Repurposing to Treat COVID-19

Drug repurposing, also known as drug repositioning, is a strategy for identifying new uses for approved or investigational drugs outside of the original medical indication. Severe acute respiratory syndrome coronavirus 2 (SARS-CoV-2) is responsible for causing coronavirus 2019 (COVID-19). The virus infects host cells by attaching the receptor-binding domain on its spike glycoprotein to the angiotensin-converting enzyme (ACE2) receptor in the host. Therefore, ACE2 is considered a potential target for the treatment of COVID-19 [22]. To identify potential repositioned drugs for COVID-19 treatment, this study predicted whether the drugs in DrugBank (totaling 11,296 drugs) could bind to ACE2 and prevent SARS-CoV-2 from entering the host cells, thus halting infection. This was achieved through CapBM-DTI, as shown in Figure 1H. Since we have models trained on three human datasets (Dataset 1, Dataset 2, and Dataset 3), we need to choose a model suitable for predicting ACE2-binding drugs based on the amount of data and the balance of ACE2 BLASTP results in a positive and negative DTI subset. We eventually choose the CapBM-DTI model, whose training set is Dataset 2, because not only does Dataset 2 have the largest amount of data (64026 samples), but also according to the BLASTP results of ACE2 aligned to Dataset 2 (Table S5), ACE2 can be blasted in positive and negative DTI sets, so Dataset 2 may be a positive and negative ACE2 balance dataset that is more suitable for predicting ACE2-binding drugs. Dataset 1 (Table S4) and Dataset 3 (Table S6) were not adopted because of the unbalanced results of BLASTP and the much smaller amount of data (6728 samples), respectively. Importantly, six COVID-19 therapeutic drugs reported in the literature shown in Table 5 were successfully predicted with high DTI possibility (0.83505136–0.9957441). We also provide an additional 50 ACE2-binding drugs with super high probability (DTI possibility > 0.9975) in Table S7, which have high application values of drug repurposing in clinical usages for treating COVID-19. To sum up, this case study proves the powerful application value of CapBM-DTI in the field of drug repurposing and virtual screening.

## 3. Discussion

To demonstrate the practicality of our model in drug development, we employed the treatment of COVID-19 as a case study, highlighting the model’s robust applicability and accuracy in drug repurposing and virtual screening. We also presented three case studies to showcase the model’s predictive performance on three key incremental problems in the drug development process: (a) predicting which compound molecules are “pan-assay interference” (PAIN) substances that bind promiscuously to many biochemical targets, producing extensive side effects or toxicity (Table S8); (b) accurately identifying a small subset of specific amino acids that are close to viable, druggable targets in protein sequences (binding region prediction) (Table S9); and (c) distinguishing binding–enhancing analogs from loss-of-potency analogs to refine activity and selectivity during lead optimization (Table S10). We believe that with the ongoing accumulation of drug-related data and the continual advancement of artificial intelligence algorithms, drug–target prediction models will play an increasingly pivotal role in the realm of drug development.

## 4. Materials and Methods

### 4.1. Experimentally Validated Datasets

Most supervised learning methods are restricted to the issue of negative sample selection due to the absence of experimentally validated non-DTI data. As a result, such approaches can only randomly select negative DTI data from unknown drug–target pairs of interest; however, these selected negative samples may comprise positive DTIs, which severely affects the selection performance and the generalization ability of the model [21,26,27,28,29,30]. To overcome this obstacle, it is most straightforward to create experimentally validated benchmark standard datasets containing both positive and negative DTI datasets (Figure 1A).

We collected two DTI datasets, Dataset 1 (medium-sized expert-curated dataset) from KinaseSARfari [31] and IUPHAR [32], and Dataset 2 (large-sized BioAssay-based dataset) from the PubChem BioAssay database [33]. For Dataset 1, KinaseSARfari contains 404 compounds and 365 proteins comprising a positive DTI dataset of 3969 with the dissociation constant (Cut off: Kd < 10 μm [34]) and an experimental validated negative dataset of 11,768. Moreover, we also obtained a positive DTI dataset of 13,643, consisting of 1541 compounds and 6218 proteins from IUPHAR. The percentages of different types of target proteins in Dataset 1 are as follows (Table S1): kinase (3.1%), enzyme (13.7%), GPCR (18.3%), ion channel (5.6%), nuclear receptor (1.7%), transporter (1.4%), and others (56.2%). For Dataset 2, we collected “Active” DTIs from the assays with the dissociation constant (Kd < 10 μm) as a positive DTI dataset. For the negative samples, we took the samples annotated as “Inactive” from the other assay types. Because the PubChem BioAssay database contained too many negative samples, we first collected only negative samples whose drug or target was included in the positive samples in the PubChem BioAssay database. Second, we randomly selected as many negative samples as positive DTIs from the PubChem BioAssay database. In this way, a total of 64,026 positive and negative samples containing 14,737 drugs and 2709 proteins were generated. In Dataset 2, the proportions of distinct target protein categories are as delineated (Table S1): kinase (5%), enzyme (45.8%), GPCR (9.2%), ion channel (0.7%), nuclear receptor (2%), transporter (0.3%), and others (37%).

In addition, we also selected two DTI datasets (i.e., Dataset 3 and Dataset 4), originally proposed by Liu et al. [21], as benchmark datasets because they are universally applicable and effective in drug discovery and development. They performed statistical tests to test the protein (or compound) corresponding to each compound (or protein) in the negative samples. As a result, their datasets contain highly credible negative samples of compound–protein pairs [19]. The detailed statistics, Venn diagram, and classification and percentage of target proteins of the 4 DTI datasets are shown in Table 1, Figure S1, and Table S1, respectively.

### 4.2. Framework of the Constructed Model

**Figure 6:**
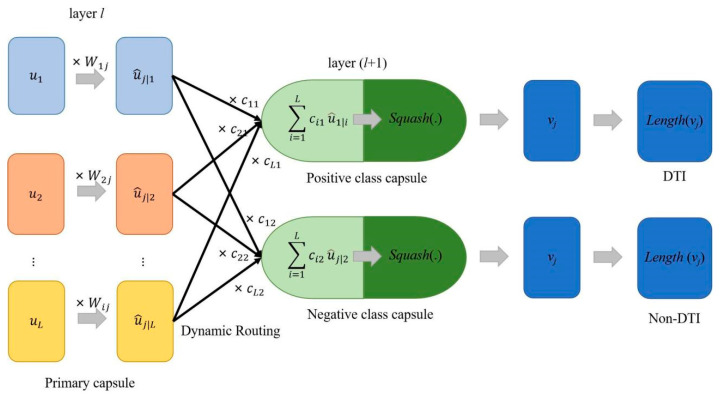
Computation process between the primary capsule layer and the class capsule layer. The prediction vector ${\hat{u}}_{j|i}$ is computed by multiplying $u_{i}$ by a transform matrix $W_{ij}$. The class capsule layer contains positive and negative capsules, each of which are calculated by a weighted sum of all prediction vectors and the squash function. During dynamic routing, the class capsule layer generates two output vectors $v_{j}$, whose length (L2 norm) denotes the probability distribution of the two classes, DTI or non-DTI.

In this article, we construct a novel capsule network-based model for DTI prediction based on large-scale pre-trained bidirectional encoder representations from transformers (BERT) and message-passing neural networks (MPNN). An overview of the DTIs model can be seen in Figure 1D, which has three modules: (a) the BERT-based protein sequence encoding module (Figure 1B), (b) MPNN-based drug molecule encoding module (Figure 1C), and (c) capsule network-based DTI decision-making module (Figure 6). Firstly, we generate embedding vectors for protein sequences in the feature engineering of the protein sequences module by utilizing the auto-encoder ProtBert model, which is pre-trained on UniRef100 data containing 216 million protein sequences. Therefore, 1024-D vectors (dimensionality of the features extracted by the ProtBert model) can be used to represent the proteins. After a fully connected layer and batch normalization layer, 1024-D vectors are transformed into 200-D vectors. Secondly, in the feature engineering of the drug molecule module, structures of drug molecules are represented by 64-D vectors through the message-passing neural network (MPNN). Lastly, the 264-D vectors (a concatenation of protein sequence feature and drug feature) are fed into the capsule network-based decision-making model to generate interaction information. The optimization module contains a cross-entropy loss. At the end of the model, we can obtain an interaction score and a non-interaction score (generated by a length layer in the capsule network); the pair is an interaction if the interaction score is bigger than the non-interaction score. 

The model in this study was constructed with the help of the TensorFlow library and the Keras framework (https://keras.io/, accessed on 15 August 2023). Overfitting was avoided by employing an early stopping strategy during model training. In particular, the training process was terminated when the accuracy did not improve within 20 epochs. The Adam optimizer was used, and the batch size and learning rate were set to 64 and 0.0001, respectively. We use grid search for hyperparameter tuning, and the optimal parameters obtained are shown in Table S2. NVIDIA A1000 40GB GPUs in high-performance computing systems were utilized throughout the training process.

#### 4.2.1. Feature Extraction from Protein

For protein feature extraction, numerous word-embedding techniques were recently utilized [35], but these techniques may map each word to its vector, making this representation context-independent. In response to the exponential growth of textual data, the first fine-tuning-based representation model, bidirectional encoder representations from transformers (BERT) [36], can generate distinct representations for the same word based on context [36,37]. In particular, Elnaggar et al. released a model named ProtBert that was trained on UniRef100 datasets that contained 216 million protein sequences [17]. The structure of ProtBert is similar to that of the original Bert publication, and the BERT model includes some unique encoding symbols, such as [CLS] and [SEP]. However, accurately predicting the complete 3D structure of a protein solely from its sequence using BERT-based models remains a complex and challenging problem [17,38,39,40,41]. While BERT-based models are not specifically designed to provide high-resolution 3D structural predictions similar to AlphaFold [42], they showed promise in capturing local structural features such as secondary structure (alpha-helices and beta-sheets), solvent accessibility, and other properties that can be inferred from sequence patterns [4,43]. In this study, we employ the ProtBert, a large protein language model, to extract contextual features from protein sequences utilizing transfer learning (Figure 1B). This approach may aid in capturing the local structure of proteins and simulating the binding interactions between drugs and the protein’s local structure.

We add the [CLS] token, which serves as an aggregate sequence representation and is typically utilized for sequence classification tasks in the BERT model. Furthermore, the [SEP] token, which is marked as *R_L+1_*, is added at the end of the protein sequence. The protein can be represented as a feature matrix *P_BERT_,* and every amino acid can be converted to a 1024-dimensional vector *BERT_Ri_* from the final layer of ProtBert.

(1) $$BERT_{R_{i}}\;=\;[BERT_{R_{i}}^{1}\;BERT_{R_{i}}^{2}\;BERT_{R_{i}}^{3}\ldots BERT_{R_{i}}^{1024}]$$

(2) $$P_{BERT}=\left[\begin{array}{ccc} BERT_{R_{0}}^{1} & \cdots & BERT_{R_{0}}^{1024}\\ \vdots & \ddots & \vdots \\ BERT_{R_{L+1}}^{1} & \cdots & BERT_{R_{L+1}}^{1024} \end{array}\right]$$

Equation (2) demonstrates that *P_BERT_* sizes vary depending on the protein length. The matrix was averaged (mean pooled) over the vertical axis to create the protein sequences in the same mathematics, and the resulting 1024-dimensional vector was referred to as BERT_Mean.

(3) $$BERT\_Mean_{n}=\frac{\sum_{I=0}^{i=L+1}BERT_{R_{i}}^{n}}{L+2}\;\left(1\leq n\;\leq 1024\right)$$

(4) $$P_{BERT\_Mean}\;=BERT\_Mean_{1}\;BERT\_Mean_{2}\ldots BERT\_Mean_{1024}$$

#### 4.2.2. Feature Extraction from Drug Molecule

The message-passing neural network (MPNN) [44] is a kind of generalized graph neural network, which is very suitable for extracting features from graph-structured data. MPNN was recently used to solve problems involving the prediction of molecular properties [45,46,47,48]. In the DTI prediction task, we employ a message passing neural network as a drug feature encoder for the atomic graph (Figure 1C).

The module takes a 2-D atomic graph G={V, E} as its input. Specifically, V is the set of nodes, containing the various atoms in the molecule V={C, H, O……}. E is the set of edges, which contains a total of four types. $E=\left\{e_{v,w}\in type|v,w\in V\right\}$, type={single, double, triple, aromatic bond}.

In particular, *V* is the collection of nodes that contain the various atoms that make up the molecule V={C, H, O……}. The set of edges known as *E* has four distinct types.

$$E=\left\{e_{v,w}\in type|v,w\in V\right\}\;type=\left\{single,\;double,\;triple,\;aromatic\;bond\right\}$$

Graph feature encoder is performed through two phases:

Phase 1: Message passing. Along the edges of the graph, nodes transmit their information in the form of message vectors to other neighbor nodes, and nodes update their hidden features by aggregating the message vectors transmitted by their neighbors. Each node receives message vectors from its K-th neighbors after K times of message passing, and the hidden features of each node are updated K times.

Phase 2: Readout. A readout function is used to combine the features of all nodes into a representation of the entire graph after the hidden features of all nodes are updated.

##### Message Passing

A fixed-size feature $h_{v}^{\left(0\right)}$∈*R^r^* of the atom’s chemical information (such as its type, valency, number of implicit H, number of electrons, hybridization type, and number of aromatic rings) is initialized by each node. The hidden features of the nodes are updated iteratively along the edges of the graph by passing information between neighbor nodes. As a result, we define the locally operated calculation step for the aggregated message vector $m_{v}^{\left(k\right)}$ as

(5) $$\;\;m_{v}^{\left(k\right)}=Aggregation\left(h_{v}^{\left(k\right)},h_{w}^{\left(k\right)},e_{v,w}|w\in N\left(v\right),e_{v,w}\in E\right)$$

where *Aggregation* is a message-aggregating function, N(v) is a self-included neighborhood of the node *v*, and $e_{v,w}$ is the edge that separates the node v from the node w.

Notably, the message vector generated by the sender node is passed to the neighbor nodes by a specific type of edge. The receiving node aggregates messages from its neighbors, including information about the neighbor nodes and edges between the nodes. Then, each node updates the hidden features using its current hidden features $h_{v}^{\left(k\right)}\;$and the message from its neighbors $m_{v}^{\left(k\right)}$. This is completed according to the following formulas:

A particular kind of edge is responsible for transferring the message vector that is generated by the sender node to the neighboring nodes. The messages sent by its neighbors are compiled by the receiving node, which also collects data about the edges that separate the nodes. Then, each node uses its current hidden features $h_{v}^{\left(k\right)}$ and the message from its neighbors $m_{v}^{\left(k\right)}$ to update the hidden features. This is completed using the following formula:

(6) $$h_{v}^{\left(k+1\right)}=GRU\left(h_{v}^{\left(k\right)},m_{v}^{\left(k\right)}\right).$$

As the update function, the gated recurrent unit (GRU) takes the most recent node state as input and updates it based on previous node states. To put it another way, the GRU gets its information from the most recent node state, while the memory state of the GRU contains information from previous nodes.

After K times of message passing, hidden features of each node contain the messages of its Kth neighbor nodes.

##### Readout

When the message-passing procedure ends, the k-step-aggregated node states are to be partitioned into subgraphs (corresponding to each molecule in the batch) and subsequently reduced to graph-level embedding. In this study, a transformer encoder + average pooling is used. Specifically:

- The k-step-aggregated node states will be partitioned into the subgraphs (corresponding to each molecule in the batch);
- Each subgraph will then be padded to match the subgraph with the greatest number of nodes;
- The (stacked padded) tensor, encoding subgraphs (each subgraph containing a set of node states), are masked to make sure the paddings do not interfere with training;
- Finally, the tensor is passed to the transformer followed by average pooling.

#### 4.2.3. Capsule Network

CNNs significantly outperformed many conventional curated feature extraction models over the past few years, achieving breakthroughs in numerous fields, such as computer vision [49] and bioinformatics [18,50,51,52,53]. CNNs have some success, but they are limited by their inability to learn spatial relationships between features and the invariance caused by pooling operations [54]. Sabour et al. proposed a novel DL theory called capsule network (CapsNet) to address these issues [55,56,57]. The fundamental component of capsule networks is the capsule, which is a collection of neurons arranged in the shape of a vector. A primary capsule layer and a class capsule layer make up the capsule layer. In contrast to conventional neural networks, capsule networks use vectors as inputs and outputs rather than scalar. Each vector (capsule) represents a type of pattern (values for feature properties such as velocity, size, orientation, and color) while the orientation of the capsule denotes the characteristics of that pattern.

A Conv1D is located in the primary capsule layer and is utilized for additional feature extraction. Multiple 8-dimension vectors are transformed from Conv1D’s outputs (the dimension is a hyper-parameter). The length of a vector, according to the idea of capsule networks, indicates the probability of the pattern that is presented. These 8-dimension vectors are treated with squash, a non-linear function that does not change the direction of the vector but compresses its length to a value between 0 and 1.

(7) $$squash\left(s\right)=\frac{{\left\|s\right\|}^{2}}{1+{\left\|s\right\|}^{2}}\frac{s}{\left\|s\right\|}$$

where the conditions are

squash(s)≈‖s‖s when s is small and

$squash\left(s\right)\approx \;\frac{s}{\left\|s\right\|}$ when s is large.

The class capsule layer contains two 16-dimensional vectors for this binary classification task: a positive capsule and a negative capsule. The input concatenate protein sequences and drug structures are represented by each capsule in this layer. The computation process between the primary and class capsule layers is depicted in Figure 2. To obtain the prediction vectors ${\hat{u}}_{j|i}$ from capsule *i* to *j*, the outputs of the primary capsule layer $u_{i}$ are first multiplied by the weight matrix $W_{ij}$, a learnable weight matrix. The weighted sum of all ${\hat{u}}_{j|i}$ determined by the following equation is $S_{j}$.

(8) $${\hat{u}}_{j|i}=W_{ij}u_{i}$$

(9) $$S_{j}=\sum_{i=1}^{L}c_{ij}{\hat{u}}_{j|i}$$

where $c_{ij}$ is coupling coefficients determined by the dynamic routing process through comparing the prediction ${\hat{u}}_{j|i}$ and real output *v_j_* and reflects the probabilities of primary capsules that should be coupled to the class capsules activated by the specific input target sequences and drug structures. *L* denotes the number of primary capsules.

Algorithm 1 demonstrates the complete process of dynamic routing. A scalar product $b_{ij}={\hat{u}}_{j|i}^{T}v_{j}\;$is the log prior probability between primary capsule *i* and class capsule *j* and $c_{ij}$ is calculated as a softmax function on $b_{ij}$. Therefore, the sum of the coupling coefficients of primary capsule *i* to class capsules $\sum_{k=1}^{N}c_{ik\;}$equals 1, where *N* denotes the class capsule number. The number of iterations r is a hyper-parameter that needs to be assigned in advance. During dynamic routing, the class capsule layer generates two output vectors $v_{j}$. The elements of $v_{j}$ encode the features whose length (L2 norm) denotes the probability distribution of the two classes, DTI or non-DTI. Therefore, the final part of the network aims to calculate its length, which is calculated as follows:
**Algorithm 1:** Dynamic Routing**Input:** ${\hat{u}}_{j|i}$, *r* **and** *l* **Output:** *v_j_*for all capsule *i* (primary capsule) in layer *l* and capsule *j* (class capsule) in layer (*l* + 1):*b_ij_* ⇐ 0for *r* iterations dofor all capsules *i* in layer *l*: *c_i_* ⇐ softmax(*b_i_*)for all capsules *j* in layer (*l*+1): *s_j_* ⇐ $\sum_{i}c_{ij}{\hat{u}}_{j|i}$for all capsules *j* in layer (*l*+1): *v_j_* ⇐ squash(*s_j_*)for all capsule *i* in layer *l* and capsule *j* in layer (*l*+1):*b_ij_* ⇐ *b_ij_* +${\hat{u}}_{j|i}v_{j}\;$end forreturn *v_j_*

The entire dynamic routing process is shown in Algorithm 1. A scalar product $b_{ij}={\hat{u}}_{j|i}^{T}v_{j}\;$is the log prior probability between primary capsule *i* and class capsule *j*, and the softmax function on $b_{ij}$ is used to calculate $c_{ij}$. As a result, the sum of the coupling coefficients of primary capsule *i* to class capsules $\sum_{k=1}^{N}c_{ik\;}$equals 1, where *N* denotes the class capsule number. The number of iterations r is a hyper-parameter that needs to be predetermined. During dynamic routing, the class capsule layer creates two result vectors $v_{j}$. The elements of $v_{j}$ encode the features whose length (L2 norm) indicates the probability distribution of the two classes, DTI or non-DTI. Therefore, the final section of the network aims to calculate its length as follows:

(10) $$p_{j}=\left\|v_{j}\right\|{}_{2}$$

Finally, this study utilizes the margin loss introduced by Sabour et al. [56], which is calculated as follows for DTI and non-DTI classes:

(11) $$L_{c}=I_{c}\max{\left(0,m^{+}-{\left\|v_{c}\right\|}_{2}\right)}^{2}+\lambda (1-I_{c})\max{\left(0,{\left\|v_{c}\right\|}_{2}-m^{-}\right)}^{2}$$

where *c* and I denote the classification category and the indicator function, respectively. In particular, $I_{c}=1$ if the current sample belongs to class c; otherwise $I_{c}=0$. $m^{+}$, $m^{-}$, and λ are hyper-parameters in this function, and we use the suggested values 0.9, 0.1, and 0.5, respectively.

### 4.3. Performance Metrics

The determination of a pair belonging to an interactive drug–target pair or non-interactive drug–target pair is in the case of single-label classification. The metrics such as sensitivity (Sen.), specificity (Spe.), precision (Pre.), accuracy (Acc.), and the F1 measure (F1) are frequently used. The specific formulas are as follows:

(12) $$\mathrm{Sen}.=\frac{\mathrm{TP}}{P}$$

(13) $$\mathrm{Spe}.=\frac{\mathrm{TN}}{N}$$

(14) $$\mathrm{Pre}.=\frac{\mathrm{TP}}{\mathrm{TP}+\mathrm{FP}}$$

(15) $$\mathrm{Acc}.=\frac{\mathrm{TP}+\mathrm{TN}}{P+N}$$

(16) $$F1=\frac{2\mathrm{Sen}*\mathrm{Pre}}{\mathrm{Sen}+\mathrm{Pre}}$$

where TP is true positive, TN is true negative, FP is false positive, FN is false negative, T is positive, and N is negative.

Additionally, the precision recall (PR) curve and the receiver operating characteristic (ROC) curve are frequently utilized to intuitively assess a predictor’s overall predictive performance. In this section, we evaluated the predictive performance in general by calculating the area under the ROC curve (auROC) and the area under the PR curve (auPRC). The auROC and auPRC values range from 0.5 to 1. The higher their values are, the better the model performance is.

## 5. Conclusions

In this study, we developed a potent and robust capsule-based drug–target interaction prediction framework named CapBM-DTI based on drug structures and protein sequences. According to our knowledge, this study is the first to use a capsule network to classify drug–target interactions. Specifically, we attempted to use pre-trained BERT to present proteins through transfer learning that map each word (amino acids are considered as words) to a potential vector space where geometric relationships can be applied to characterize semantic relationships. Additionally, we convert SMILES into an atomic 2D graph to represent drug and employ the message-passing neural network (MPNN) to extract the graph’s high-order structures as well as semantic relations. Lastly, we adopted a capsule network, which describes the internal hierarchical representation of features as a classifier to differentiate DTI and non-DTI. Importantly, we constructed two experimentally validated datasets to get over the drawback that negative DTI data from other research are picked at random from unidentified drug–target combinations. Furthermore, CapBM-DTI achieved satisfactory robust performance and strong generalizability compared to SOTA methods on seven experimentally validated DTI datasets with varying amounts of data, including human (*Homo sapiens*) and worm (*Caenorhabditis elegans*) species datasets. The case studies demonstrates the applicability of the model in virtual screening, drug repositioning and drug development. Overall, CapBM-DTI is a robust and accurate DTI prediction framework, which offers more significant potential and scope for drug target identification, virtual screening, drug repurposing, and drug development. The source code and data are freely available at https://github.com/huangyixian666/CapBM-DTI (accessed on 15 August 2023).

## Figures and Tables

**Table 1:** Statistics of DTI datasets.

Dataset	Dataset 1	Dataset 2	Dataset 3 [21]	Dataset 4 [21]
Species	*H. sapiens*	*H. sapiens*	*H. sapiens*	*C. elegans*
The number of compounds	6602	14,737	2726	1767
The number of proteins	1900	2709	2001	1876
The number of positive interactions ^a^	16,627	32,013	3364	3893
The number of negative interactions ^b^	11,768	32,013	3364	3893
Density (%)	0.226	0.16	0.123	0.235

^a^: Positive interactions are experimentally validated drug–target interactions. ^b^: Negative interactions are experimentally validated non-drug–target interactions.

**Table 2:** Comparison of performances between our model and previous models from four datasets (training dataset: test dataset = 8:2). Best performance values are in bold.

Model	Dataset 1				Dataset 2				Dataset 3				Dataset 4			
	Accuracy	F1	AUC	AUPR	Accuracy	F1	AUC	AUPR	Accuracy	F1	AUC	AUPR	Accuracy	F1	AUC	AUPR
DeepConv-DTI	0.877	0.894	0.941	0.964	0.825	0.843	0.933	0.932	0.611	0.662	0.636	0.780	**0.943**	0.936	0.978	0.975
CPI_prediction	0.885	0.894	0.943	0.967	0.864	0.852	0.935	0.938	0.891	0.901	0.936	0.945	0.926	0.931	0.965	0.972
TransformerCPI	0.872	0.883	0.940	0.965	0.855	0.851	0.938	0.935	0.892	0.893	0.954	0.958	0.914	0.911	0.977	0.977
IIFDTI	0.857	0.879	**0.946**	0.968	0.736	0.631	**0.952**	0.943	0.880	0.890	0.951	**0.963**	0.938	0.942	0.980	**0.983**
**CapBM-DTI**	**0.893**	**0.901**	**0.946**	**0.970**	**0.87**	**0.862**	0.935	**0.944**	**0.915**	**0.915**	**0.958**	0.961	0.941	**0.938**	**0.982**	**0.983**

**Table 3:** Statistics of new compound subset, new protein subset, and new pairs subset from Dataset 3.

Dataset	New Compounds Dataset ^c^		New Proteins Dataset ^d^		New Pairs Dataset ^e^	
	Training Set	Test Set	Training Set	Test Set	Training Set	Test Set
The number of compounds	2000	726	2406	824	1770	227
The number of proteins	1797	915	1500	501	1351	125
The number of positive interactions ^a^	2569	795	2888	476	2218	125
The number of negative interactions ^b^	2445	919	2532	832	1834	221
Density (%)	0.140	0.258	0.150	0.317	0.169	0.674

^a^: Positive interactions are experimentally validated drug–target interactions. ^b^: Negative interactions are experimentally validated non-drug–target interactions. ^c^: There were no intersections of compounds in the training set and compounds in the test set. ^d^: There were no intersections of proteins in the training set and proteins in the test set. ^e^: There were no overlaps between the training and test datasets. Neither the training compound nor the training protein appeared in the test set.

**Table 4:** Comparison of the proposed method with the previous model in terms of three settings. Best performance values are in bold.

Model	New Compounds				New Proteins				New Pairs			
	Accuracy	F1	AUC	AUPR	Accuracy	F1	AUC	AUPR	Accuracy	F1	AUC	AUPR
DeepConv-DTI	0.744	0.714	0.808	0.699	0.792	0.719	0.849	0.668	0.668	0.590	0.729	0.645
CPI_prediction	0.571	0.613	0.599	0.669	0.399	0.462	0.443	0.53	0.455	0.567	0.527	0.645
TransformerCPI	0.779	0.743	**0.847**	0.832	0.829	0.744	0.892	0.841	0.723	0.575	0.753	0.661
IIFDTI	0.750	0.765	0.834	0.818	0.851	0.814	0.922	0.912	0.705	0.608	**0.765**	0.668
**CapBM-DTI**	**0.788**	**0.766**	0.832	**0.848**	**0.906**	**0.874**	**0.945**	**0.908**	**0.725**	**0.615**	0.751	**0.704**

**Table 5:** Reference-supported ACE2-binding drugs identified through DrugBank large-scale virtual screening.

DrugBank ID	Name	Interact Status ^a^	Non-DTI Possibility	DTI Possibility	Drug Mechanism of Action	Ref
DB00691	Moexipril	1	0.03831145	0.98414844	An angiotensin-converting enzyme inhibitor (ACE inhibitor) used for the treatment of hypertension and congestive heart failure	[23]
DB00477	Chlorpromazine	1	0.01079374	0.995042	Facilitating ACE2 endocytosis, reducing virus-receptor binding capacity in vitro	[23]
DB13609	Umifenovir	1	0.12442836	0.84877896	Decreased viral endocytosis	[24]
DB12466	Favipiravir	1	0.03333556	0.98836476	Decreased viral endocytosis	[24]
DB13729	Camostat	1	0.0929359	0.83505136	TMPRSS2 hydrolyzes ACE2 and thus degrades ACE2	[25]
DB12598	Nafamostat	1	0.04878383	0.9957441	TMPRSS2 hydrolyzes ACE2 and thus degrades ACE2	[25]

^a^: 1 means interact while 0 means non-interact.

## Data Availability

CapBM-DTI and datasets of this study are available at https://github.com/huangyixian666/CapBM-DTI (accessed on 15 August 2023).

## Supplementary Materials

The following supporting information can be downloaded at: https://www.mdpi.com/article/10.3390/ijms241814061/s1. References [58,59,60,61] are cited in the supplementary materials.
