# Supplementary material for: A Robust Drug–Target Interaction Prediction Framework with Capsule Network and Transfer Learning
Source: Int J Mol Sci. 2023 Sep 14;24(18):14061. doi: 10.3390/ijms241814061 (PMC10531393; doi:10.3390/ijms241814061)
Supplement: Supplementary file 1 [file ijms-24-14061-s001.zip › ijms-2553792-supplementary.pdf]

Supplementary

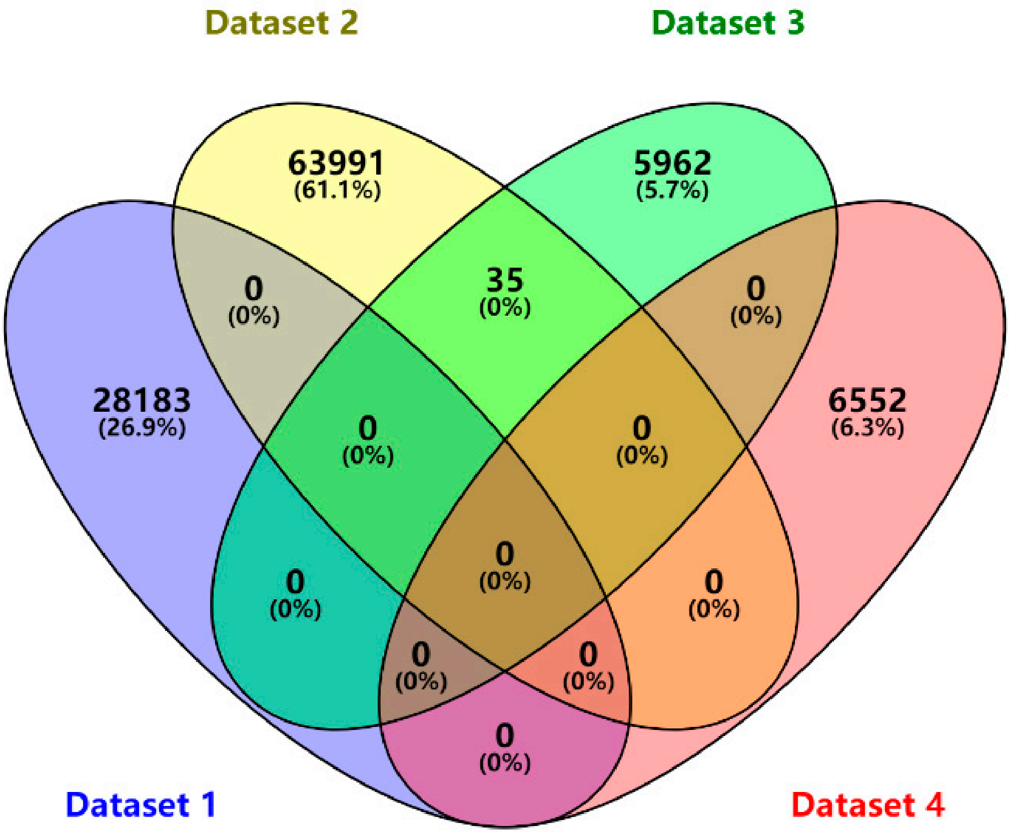

Figure S1. Venn diagram of the four DTI datasets.

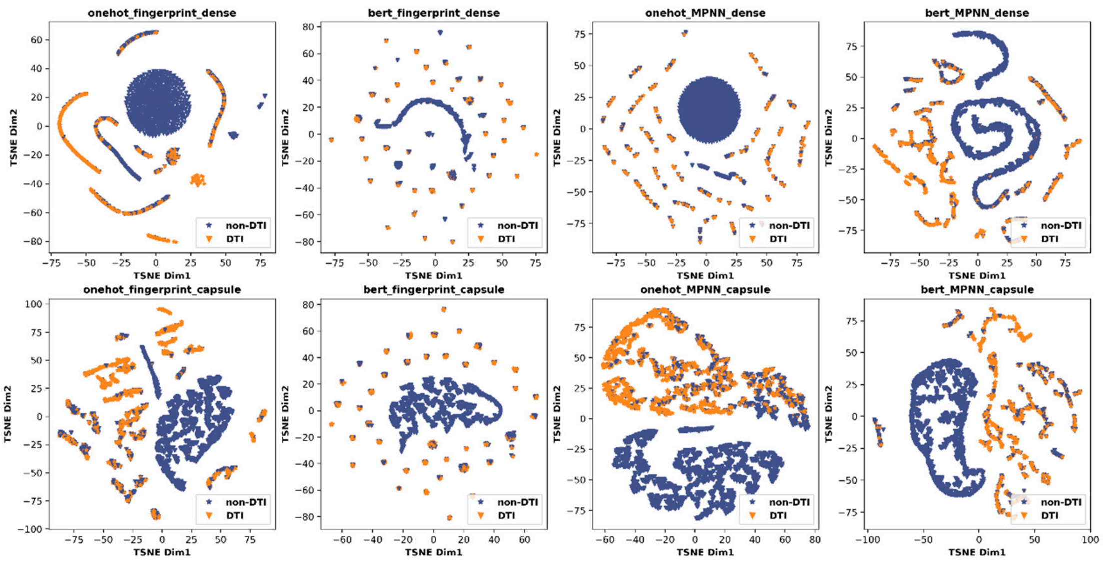

Figure S2. t-SNE visualizations of the proposed model and 7 baseline models on Dataset 1.

Table S1 Classification and percentage of target proteins in 4 datasets

Dataset	Species	Kinase	Enzyme	GPCR	Ion channel	Nuclear receptor	Transporter	Others	Sum
Dataset 1	Human	883 (3.1%)	3887 (13.7%)	5196 (18.3%)	1604 (5.6%)	483 (1.7%)	402 (1.4%)	15940 (56.2%)	28395 (100%)
Dataset 2	Human	3185 (5%)	29298 (45.8%)	5896 (9.2%)	363 (0.7%)	1322 (2%)	234 (0.3%)	23728 (37%)	64026 (100%)
Dataset 3	Human	300 (4.5%)	1917 (28.5%)	584 (8.7%)	290 (4.3%)	136 (2%)	315 (4.6%)	3186 (47.4%)	6728 (100%)
Dataset 4	Worm	Worm species do not have related data on the classification of target protein,							7786 (100%)

Table S2 Hyperparameters settings for CapBM-DTI

Hyperparameters	Search space	Best parameters
target_dense	[200,400]	200
routings	[3,6]	3
kernel_size	[5,10]	5
message_units	-	64
message_steps	-	4
num_attention_heads	-	8
dense_units	-	512
batch_size	-	64
epoch	-	1000
loss	-	binary entropy
optimizer	-	Adam

Table S3. Comparison of performances between our models and baseline model on Dataset 1

Model	Sensitivity	Specificity	Precision	Accuracy	F1	AUC	AUPR
Onehot+fingerprint+Dense	0.871	0.792	0.855	0.838	0.863	0.946	0.967
Bert+fingerprint+Dense	0.826	**0.975**	**0.979**	0.888	0.896	0.941	0.967
Onehot+fingerprint+Capsule	0.841	0.944	0.955	0.884	0.894	0.944	0.969
Bert+fingerprint+Capsule	0.827	0.974	0.978	0.888	0.896	0.921	0.959
Onehot+MPNN+Dense	0.767	0.965	0.970	0.847	0.857	0.901	0.948
Bert+MPNN+Dense	0.828	0.950	0.960	0.877	0.889	0.938	0.966
Onehot+MPNN+Capsule	**0.874**	0.844	0.891	0.862	0.883	0.927	0.962
**Bert+MPNN+Capsule**	0.836	0.973	0.978	**0.893**	**0.901**	**0.946**	**0.97**

Table S4. BLASTP Result of ACE2 (Database: Dataset 1)

Dataset	Sequences producing significant alignments	Score (Bits)	E Value
Protein sequences in positive DTI Dataset	10945	1685	0
	9851	1685	0
	11003	509	5.00E-164
	9872	509	5.00E-164
	9870	509	5.00E-164
	9804	509	5.00E-164
	9802	509	5.00E-164
	9768	509	5.00E-164
	9747	509	5.00E-164
	9744	509	5.00E-164
	9742	509	5.00E-164
	9738	509	5.00E-164
	9736	509	5.00E-164
	9733	509	5.00E-164
	9731	509	5.00E-164
	8787	509	5.00E-164
	14419	33.9	0.3
	13979	33.9	0.3
	13932	33.9	0.3
	13660	33.9	0.3
	13509	33.9	0.3
	12194	33.9	0.3
	11763	33.9	0.3
	11352	33.9	0.3
	11141	33.9	0.3
	11116	33.9	0.3
Protein sequences in negative DTI Dataset	No hits found		

Table S5. BLASTP Result of ACE2 (Database: Dataset 2)

Dataset	Sequences producing significant alignments	Score (Bits)	E Value
Protein sequences in positive DTI Dataset	1880	27.7	8.3
Protein sequences in negative DTI Dataset	31532	33.9	0.81
	31355	33.9	0.81
	31352	3.39E+01	0.81
	30430	3.39E+01	0.81
	29682	3.39E+01	0.81
	29454	3.39E+01	0.81
	28918	3.39E+01	0.81
	28503	3.39E+01	0.81
	26658	3.39E+01	0.81
	25994	3.39E+01	0.81
	19602	3.39E+01	0.81
	18090	3.39E+01	0.81
	17249	3.39E+01	0.81
	13818	3.39E+01	0.81
	12839	3.39E+01	0.81
	12004	3.39E+01	0.81
	10931	33.9	0.81
	10420	33.9	0.81
	9576	33.9	0.81
	8730	33.9	0.81
	8399	33.9	0.81
	5185	33.9	0.81
	3803	33.9	0.81
	3082	33.9	0.81
	3070	33.9	0.81
	2779	33.9	0.81
	1466	33.9	0.81
	987	33.9	0.81
	12191	33.9	0.89
	7918	33.9	0.89
	7158	33.9	0.89
	5380	33.9	0.89
	4198	33.9	0.89
	4063	33.9	0.89
	3640	33.9	0.89
	3450	33.9	0.89
	3436	33.9	0.89

3200	33.9	0.89
3183	33.9	0.89
2681	33.9	0.89
1995	33.9	0.89
926	33.9	0.89
525	33.9	0.89
387	33.9	0.89

Table S6. BLASTP Result of ACE2 (Database: Dataset 3)

Dataset	Sequences producing significant alignments	Score (Bits)	E Value
Protein sequences in positive DTI Dataset	2564	1685	0
	2423	33.9	0.08
	1416	3.39E+01	0.08
	1332	3.39E+01	0.08
	518	3.39E+01	0.08
	2236	2.77E+01	6.2
	145	2.77E+01	6.2
Protein sequences in negative DTI Dataset	3254	1685	0
	1804	1685	0
	1274	1.69E+03	0
	762	1.69E+03	0
	3322	5.09E+02	1.00E-164
	3356	2.81E+01	3.6
	3282	2.81E+01	3.6
	3156	2.81E+01	3.6
	2238	2.81E+01	3.6
	1952	2.81E+01	3.6
	1851	2.81E+01	3.6
	1635	2.81E+01	3.6
	1339	2.81E+01	3.6
	963	2.81E+01	3.6

Table S7. Drugs that interact with ACE2 with high confidence (DTI possibility > 0.9975)

DrugBank ID	Name	Drug Groups	Interact status (1: interact; 0: non-interact)	non-DTI possibility	DTI possibility
DB08152	{(2S)-1-[N-(tert-butoxycarbonyl)glycyl]pyrrolidin-2-yl}methyl (3-chlorophenyl)acetate	experimental	1	0.01591295	0.9978174
DB00419	Miglustat	approved	1	0.01230913	0.9978156
DB03461	Nicotinamide adenine dinucleotide phosphate	experimental	1	0.04863215	0.997803
DB03190	6-o-Capryloylsucrose	experimental	1	0.00750221	0.99778444
DB13106	Glycovir	investigational	1	0.02317203	0.99777514
DB07413	5'-S-[2-(decylamino)ethyl]-5'-thioadenosine	experimental	1	0.01320679	0.99775714
DB09269	Phenylacetic acid	approved	1	0.02533052	0.99775684
DB14085	Cadmium	experimental	1	0.02041934	0.99773604
DB08132	5-hydroxynaphthalene-1-sulfonamide	experimental	1	0.06120944	0.99770105
DB00302	Tranexamic acid	approved	1	0.01589582	0.9976981
DB07834	N-(cyclopropylmethyl)-2'-methyl-5'-(5-methyl-1,3,4-oxadiazol-2-yl)biphenyl-4-carboxamide	experimental	1	0.0054048	0.9976975
DB12721	RO-5028442	investigational	1	0.02743089	0.9976842
DB07569	CIS-4-METHYL-N-[(1S)-3-(METHYLSULFANYL)-1-(PYRIDIN-4-	experimental	1	0.00902602	0.9976576

	YLCARBAMOYL)PR OPYL]CYCLOHEXAN ECARBOXAMIDE				
DB09256	Tegafur	approved; investigational	1	0.0121 8555	0.9976 538
DB13209	Bismuth subnitrate	approved	1	0.0130 459	0.9976 4323
DB15424	Bfpet F-18	investigational	1	0.0310 801	0.9976 42
DB04800	1-METHYL-3-PHENYL-1H-PYRAZOL-5-YLSULFAMIC ACID	experimental	1	0.0182 131	0.9976 397
DB08595	4-[(1S,2R,5S)-4,4,8-TRIMETHYL-3-OXABICYCLO[3.3.1]N ON-7-EN-2-YL]PHENOL	experimental	1	0.0074 7552	0.9976 3715
DB03338	Heptyl glucoside	experimental	1	0.0089 1036	0.9976 3316
DB07215	GW-590735	investigational	1	0.0195 1079	0.9976 1057
DB08808	Bupranolol	experimental	1	0.0144 6043	0.9976 1015
DB08913	Radium Ra 223 dichloride	approved; investigational	1	0.0084 8691	0.9976 066
DB07381	(S)-atrolactic acid	experimental	1	0.0408 9671	0.9976 06
DB02316	1-(5-Carboxypentyl)-5-[(2,6-Dichlorobenzyl)Oxy]-1 H-Indole-2-Carboxylic Acid	experimental	1	0.0352 9239	0.9976 012
DB04026	Pseudotropine	experimental	1	0.0055 0823	0.9975 946
DB01948	1-(2,6-Dichlorophenyl)-5-(2,4-Difluorophenyl)-7-Piperidin-4-Yl-3,4-Dihydroquinolin-2(1h)-One	experimental	1	0.0068 3042	0.9975 814
DB03916	4-{2-[4-(2-Aminoethyl)Piperazin-1-Yl]Pyridin-4-Yl}-N-(3-Chloro-4-	experimental	1	0.0093 3818	0.9975 7725

	Methylphenyl)Pyrimidin-2-Amine				
DB02479	(R)-N-(3-Indol-1-Yl-2-Methyl-Propyl)-4-Sulfamoyl-Benzamide	experimental	1	0.01205768	0.9975734
DB06697	Artemether	approved	1	0.01255393	0.99757123
DB12767	Gaxilose	approved; investigational	1	0.02553476	0.99755937
DB05549	INO-1001	investigational	1	0.00744648	0.9975574
DB00241	Butalbital	approved; illicit	1	0.00797347	0.9975512
DB15414	CM-4307	investigational	1	0.01390884	0.99754983
DB09136	Isosulfan blue	approved	1	0.00844891	0.99754715
DB03322	Dexpropranolol	experimental	1	0.01450519	0.9975468
DB15687	Tridecactide	investigational	1	0.00813702	0.9975441
DB06569	P-57AS3	investigational	1	0.00864029	0.99754375
DB07757	(9aS)-4-bromo-9a-butyl-7-hydroxy-1,2,9,9a-tetrahydro-3H-fluoren-3-one	experimental	1	0.0092816	0.9975422
DB07693	N-(3,5-dibromo-4-hydroxyphenyl)-2,6-dimethylbenzamide	experimental	1	0.01542644	0.99754137
DB01936	alpha-D-arabinofuranose	experimental; investigational	1	0.01028795	0.9975381
DB06622	7-beta-Hydroxyepiandrosterone	investigational	1	0.01116393	0.99753755
DB06881	(1Z)-2-HYDROXY-3-OXOHEX-1-EN-1-YL DIHYDROGEN PHOSPHATE	experimental	1	0.00732605	0.99753374
DB16032	GW810781	investigational	1	0.01277663	0.99752307
DB13425	Flutrimazole	experimental	1	0.04378571	0.9975219
DB12794	p-Quaterphenyl	investigational	1	0.008172	0.9975169

DB08702	2,5-DIPHENYLFURAN-3,4-DICARBOXYLIC ACID	experimental	1	0.01216486	0.99751276
DB06876	N-{5-[4-(4-METHYLPIPERAZIN-1-YL)PHENYL]-1H-PYRROLO[2,3-B]PYRIDIN-3-YL}NICOTINAMIDE	experimental	1	0.01772101	0.9975081
DB13257	Ferrous sulfate anhydrous	approved	1	0.00920453	0.9975042
DB08077	2-[4-({[(3,5-DICHLOROPHENYL)AMINO]CARBONYL}AMINO)PHENOXY]-2-METHYLPROPANOIC ACID	experimental	1	0.01791428	0.9975041
DB15872	L-alpha-Glycerophosphorylethanolamine	experimental	1	0.03022476	0.997502

Table S8. Prediction results of PAIN substances and FDA-approved drugs with 20422 human proteins

Molecule type	Drug Name	PubChem Compound ID	Number of DTI	Number of non-DTI
"pan-assay interference" (PAIN) substances [1]	gossypol	3503	20422	0
	droxidopa	92974	20422	0
	menadione	4055	20422	0
	daunorubicin	30323	20422	0
FDAapproved Drug [2]	sorafenib	216239	1023	19399
	lenvatinib	9823820	1043	19379
	regorafenib	11167602	1052	19370
	cabozantinib	25102847	1054	19368

To showcase the model's ability to distinguish drug-like molecules, we select four "pan-assay interference" (PAIN) substances and four FDA-approved drugs from the literature [2,3]. We then used our CapBM-DTI model (trained on Dataset 2) to predict the binding possibility between these eight drugs and all human proteins collected from Swiss-Prot. The results are presented in **Supplementary Table S8**. Based on the findings in Table S8, our model exhibits a certain level of discriminative performance. Specifically, PAIN substances can bind to all proteins, while FDA-approved drugs can only bind to a small number of proteins.

Table S9. Model prediction results of 2-(2,4-dichlorophenoxy)-5-(hydroxymethyl)phenol (PubChem Compound ID: 23656595) binding to binding region and non-binding regions of Transthyretin (Uniprot ID: P00375)

Drug and Protein	Region	Sequence index [Start, End]	Sequence	DTI possibility	non-DTI possibility	DTI Status
2-(2,4-dichlorophenoxy)-5-(hydroxymethyl)phenol (PubChem Compound ID: 23656595) and Transthyretin (Uniprot ID: P00375) [3]	Full sequence	[1,147]	MASHRLLLLCLAGLVFVSEAGPTGTGESKCPLMVKVLDAVRGSPAINVAVHVFRKAADDTWEPFASGKTSESGELHGLTTEEEFVEGIYKVEIDTKSYWKALGISPFHEHAEVVFTANDSGPRRYTIAALLSPYSYSTTAVVTNPKE	0.8723520636558533	0.586342573165 8936	1
	Binding region	[28, 43]	SKCPLMVKVLDAVRGS	0.968401849269866	0.147558823227882	1
		[69, 78]	TSESGELHGL	0.968401849269866	0.147706046700477	1
		[108, 117]	HEHAEVVFTA	0.968391358852386	0.147521197795867	1
		[121, 147]	GPRRYTIAALLSPYSYSTTAVVTNPKE	0.968401610851287	0.147706136107444	1
	Non-binding region	[1,27]	MASHRLLLLCLAGLVFVSEAGPTGTGE	0.645132422447204	0.987516880035 4	0
		[44,68]	PAINVAVHVFRKAADDTWEPFASGK	0.645219445228576	0.987514972686767	0
		[79,107]	TTEEEFVEGIYKVEIDTKSYWKALGISPF	0.645219087600708	0.987515211105346	0
		[118,120]	NDS	0.430580437183 38	0.279024273157119	1

As a case study, we utilized the full-length sequence, binding sequence and non-binding sequence of Transthyretin (Uniprot ID: P00375) binding to 2-(2,4-

dichlorophenoxy)-5-(hydroxymethyl)phenol (PubChem Compound ID: 23656595) [3] to assess the model's ability to identify specific amino acids that are in close proximity to viable and druggable targets. **Supplementary Table S9** demonstrates that, with the exception of the short sequence "NDS" in the non-binding region (which leads to inaccurate model prediction), the model accurately predicts whether the other sequences bind to the compound, which showcases the discriminative performance of our proposed model (trained on Dataset 2) in this task.

Table S10. Model prediction results of (S)-blebbistatin analogs

Drug Name	PubChem Compound ID	IC50 (μM)	DTI possibility	non-DTI possibility	DTI Status	Structure
N-[3-[(3aS)-3a-hydroxy-6-methyl-4-oxo-2,3-dihydropyrrolo[2,3-b]quinolin-1-yl]phenyl]propanamide	145954681	<0.02	0.7770342826843262	0.4666823446750641	1	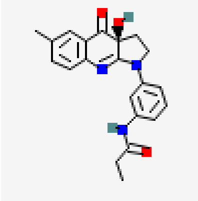
(3aS)-3a-hydroxy-6-methyl-1-naphthalen-2-yl-2,3-dihydropyrrolo[2,3-b]quinolin-4-one	145955716	<0.02	0.7468130588531494	0.6139199137687683	1	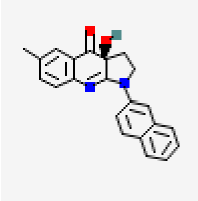
(3aS)-1-(2,3-dihydro-1H-indol-6-yl)-3a-hydroxy-6-methyl-2,3-dihydropyrrolo[2,3-b]quinolin-4-one	145973596	0.11	0.7070441246032715	0.4478471279144287	1	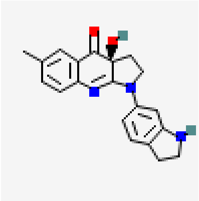
(3aS)-3a-hydroxy-1-(1H-indol-6-yl)-6-methyl-2,3-dihydropyrrolo[2,3-b]quinolin-4-one	145971197	0.12	0.7852638959884644	0.389826059341430 66	1	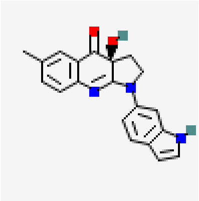
(3aS)-3a-hydroxy-6-methyl-1-phenyl-2,3-dihydropyrrolo[2,3-b]quinolin-4-one	5287792	1	0.5900148153305054	0.4920700490474701	1	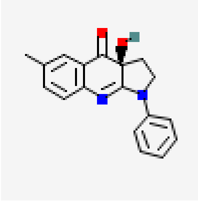

(3aS)-3a-hydroxy-6-methyl-1-(4-prop-2-enoxyphenyl)-2,3-dihydropyrrolo[2,3-b]quinolin-4-one	145951386	2.3	0.5935986638069153	0.491857290267944 34	1	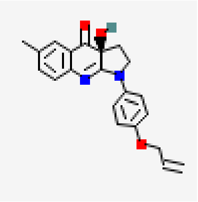
(3aS)-3a-hydroxy-6-methyl-1-(4-nitrophenyl)-2,3-dihydropyrrolo[2,3-b]quinolin-4-one	102361739	2.5	0.3495916724205017	0.2939663529396057	1	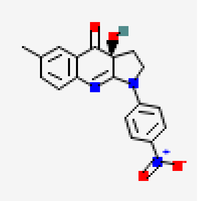

(S)-blebbistatin (Compound CID: 5287792) is a widely used compound in the study of myosin II (UniprotID: Q9UKX2), an important regulator of many motor disorders. Although its potency is too low to be clinically significant, identifying analogs with enhanced potency could provide insights for targeted drug therapy. In this study [4], the compound was modified, and the IC50 value against the target myosin II was determined. We utilized our CapBM-DTI model (trained on Dataset 2) to predict the binding possibility of these compounds to target proteins myosin II and obtained **Supplementary Table S10**. The result in **Table S10** demonstrate that the DTI possibilities predicted by our model strongly correlate with IC50 value, indicating the practicality of our model in the lead optimization.

Reference

1. Baell JB. Feeling nature's PAINS: natural products, natural product drugs, and pan assay interference compounds (PAINS), Journal of natural products 2016;79:616-628.
2. Rimassa L. Drugs in development for hepatocellular carcinoma, Gastroenterology & hepatology 2018;14:542.
3. Lee I, Nam H. Sequence-based prediction of protein binding regions and drug–target interactions, Journal of cheminformatics 2022;14:1-15.
4. Verhasselt S, Roman BI, Bracke ME et al. Improved synthesis and comparative analysis of the tool properties of new and existing D-ring modified (S)-blebbistatin analogs, Eur J Med Chem 2017;136:85-103.
